# Polyphenols from marine brown algae target radiotherapy-coordinated EMT and stemness-maintenance in residual pancreatic cancer

**DOI:** 10.1186/s13287-015-0173-3

**Published:** 2015-09-22

**Authors:** Sheeja Aravindan, Satish Kumar Ramraj, Somasundaram T. Somasundaram, Terence S. Herman, Natarajan Aravindan

**Affiliations:** Department of Marine Sciences, Center of Advance Study in Marine Biology, Annamalai University, Parangipettai, TN 608 502 India; Stephenson Cancer Center, 975 NE 10th Street, Oklahoma City, OK 73104-5419 USA; Department of Radiation Oncology, University of Oklahoma Health Sciences Center, 940 Stanton L. Young Boulevard, Oklahoma City, OK 73104 USA

## Abstract

**Introduction:**

Therapy-associated onset of stemness-maintenance in surviving tumor-cells dictates tumor relapse/recurrence. Recently, we recognized the anti-pancreatic cancer (PC) potential of seaweed polyphenol manifolds and narrowed down three superior drug-deliverables that could serve as adjuvants and benefit PC cure. Utilizing the PC- cancer stem cells (PC-CSCs) grown *ex vivo* and mouse model of residual-PC, we investigated the benefits of seaweed polyphenols in regulating stemness-maintenance.

**Methods:**

ALDH^+^CD44^+^CD24^+^ PC-CSCs from Panc-1, Panc-3.27, MiaPaCa-2, or BxPC-3 cells-derived xenografts grown *ex vivo* were either mock-irradiated, exposed to fractionated irradiation (FIR, 2Gy/D for 5 days), treated with polyphenols (100 μg/ml) of *Hormophysa triquerta* (HT-EA), *Spatoglossum asperum* (SA-EA) or *Padina tetrastromatica* (PT-EA) with/without FIR were examined for cell viability, transcription of 93 stem-cell-related molecules (QPCR profiling). Polyphenol-dependent regulation of FIR-transactivated *Oct4*, *Zic3*, *EIF4C*, *Nanog,* and *LIF* (QPCR) and functional translation of Nanog, SOX2, and OCT3/4 (immunoblotting) were examined in Panc-1/Panc-3.27/MiaPaCa-2/BxPC-3-xenografts derived PC-CSCs. Effect of seaweed-polyphenols in the regulation of EMT (N-Cadherin), pluripotency- (SOX2, OCT3/4, Nanog) and stemness-maintenance (PI3KR1, LIF, CD44) in therapy (FIR, 2Gy/D for 5D/wk for 3-weeks) resistant residual tumors were examined by tissue microarray construction and automated immunohistochemistry.

**Results:**

*Ex vivo* exposure of PC-CSCs to SA-EA, PT-EA and HT-EA exhibit dose-dependent inhibition of cell viability. FIR amplified the transcription of 69, 80, 74 and 77 stem-cell related genes in MiaPaCa-2-, Panc-1-, Panc-3.27- and BXPC3-established xenograft-derived ALDH^+^CD44^+^CD24^+^PC-CSCs. Treatment with SA-EA, PT-EA, or HT-EA completely suppressed FIR-activated stem-cell transcriptional machinery in ALDH^+^CD44^+^CD24^+^PC-CSCs established from MiaPaCa-2, Panc-1, Panc-3.27 and BXPC3 xenografts. QPCR validated *EIF4C, OCT3/4, Nanog, LIF,* and *ZIC3* transcriptional profile outcomes. Nanog, Sox2, and OCT3/4 immunoblotting affirmed the PC-CSC radiosensitizing benefit of seaweed polyphenols. Residual-PC tissues microarrayed and immunostained after *in vivo* treatments recognized complete regulation of FIR-induced SOX2, OCT3/4, Nanog, LIF, CD44, PIK3R1, N-Cadherin, and E-Cadherin with SA-EA, PT-EA, and HT-EA.

**Conclusions:**

These data, for the first time, documented the EMT/stemness-maintenance in therapy-resistant PC-CSCs. Further, the data suggest that seaweed polyphenols may inhibit PC relapse/recurrence by targeting therapy-orchestrated stem-cell signaling in residual cells.

**Electronic supplementary material:**

The online version of this article (doi:10.1186/s13287-015-0173-3) contains supplementary material, which is available to authorized users.

## Introduction

Clinical and laboratory evidence suggests that several common human cancers contain populations of rapidly proliferating clonogens that can have a substantial impact on local control following chemoradiotherapy or conventional radiotherapy [1]. Recurring tumors may arise from remnant cells of the original neoplasm that have escaped therapeutic intervention and later become visible at the original site [2, 3]. For many cancers, it has been hypothesized that tumor cells responsible for failures in long-term remission exhibit stem cell properties [4–6]. It is now being appreciated that tumors contain a small number of tumor-forming and self-renewing cancer stem cells (CSCs) within a population of nontumor-forming cancer cells that contribute to pancreatic cancer (PC) progression and relapse [7]. The CSC hypothesis suggests that conventional chemoradiotherapy kills differentiated/differentiating cells that form the bulk of the tumor, but cannot generate new cells. Tumor relapse may occur because CSCs remain untouched by treatment, suggesting that the removal of CSCs is crucial for effective therapy. In addition, recent evidence points to the existence of programmed functional plasticity not only in CSCs, but also in nonstem cancer cell populations [8, 9]. Detailed pathological analysis of PC has confirmed genetically traceable unique subclone association with metastatic lesions [10, 11], and further suggests that multiple genetic subclones are constantly evolving, competing in parallel within the primary tumor, and might independently give rise to metastatic lesions. In addition, recent genetic profiles of CSCs [12] demonstrated genetically diverse tumor-initiating cells in genetically-driven tumors. As CSCs have been shown to be more resistant to chemoradiation than the rest of the tumor cell population [13–16], this selective pressure would automatically select the genetic clones that contain a higher proportion of CSCs, and thereby have greater potential for reconstituting tumor growth once the therapeutic regimen is finished. In this regard, delineating the contribution of reactivated (after first-line therapy) developmental signaling pathways to PC initiation and progression [17] would shed light on understanding the CSCs’ role in PC progression and relapse.

Early forays into CSC-targeted therapies in combination with standard therapies [18, 19] have shown that some combinations have efficacy against PC-CSCs [20, 21], decreased tumorosphere-forming capacity, and in vivo tumorigenicity. These approaches reveal the possibility of developing CSC-targeted therapies that could potentially be used alongside chemotherapy and radiation to specifically eliminate CSC subpopulations and reduce tumor recurrence. Seaweeds rich in polyphenols [22] have been shown to exert anti-tumor [23] potential, particularly in inhibiting cell proliferation [24], tumor regression [25], and inhibition of metastasis [26]. A close association between polyphenols’ anti-carcinogenic activity and antioxidant activity has been reported in mouse models of carcinoma [25, 27]. Recent investigations demonstrated the anti-proliferative, pro-apoptotic, DNA-damaging, anti-angiogenic, growth-inhibiting, cell-cycle arrest, and anti-metastatic functions of seaweed extracts in various tumor models [28–31]. We have recently demonstrated that polarity-based polyphenol fractions extracted from brown algae exerted potent anti-PC potentials *in vitro*, and further identified three high-polarity fractions that could be clinically translatable [32]. This study defined the therapy-associated stem cell-related molecular signature in residual ALDH^+^CD44^+^CD24^+^ PC-CSCs, and further identified the clinical benefits of these fractions in this setting.

## Methods and materials

### Cell culture

Genetically diverse human Panc-1, MiaPaCa-2, Panc-3.27, and BxPC-3 cells were cultured and maintained as described earlier [32, 33].

### Xenotransplantation mouse model

All experiments conformed to American Physiological Society standards for Animal Care, were carried out in accordance with the guidelines laid down by the National Research Council, and were approved by the University of Oklahoma Health Sciences Center Institutional Animal Care and Use Committee. Seven-week-old male athymic NCr-*nu/nu* nude mice weighing 25–30 g were acclimatized for at least 3 days before the study. We administered 5 × 10^6^ Panc-1, Panc-3.27, MiaPaCa-2, or BxPC-3 cells (with 30 % Matrigel; Corning, Tewksbury, MA, USA) subcutaneously into their right flanks. Tumor growth was periodically monitored and was allowed to grow to a volume of at least 100 mm^3^.

### Isolation of PC-CSCs

**Fig. 1:**
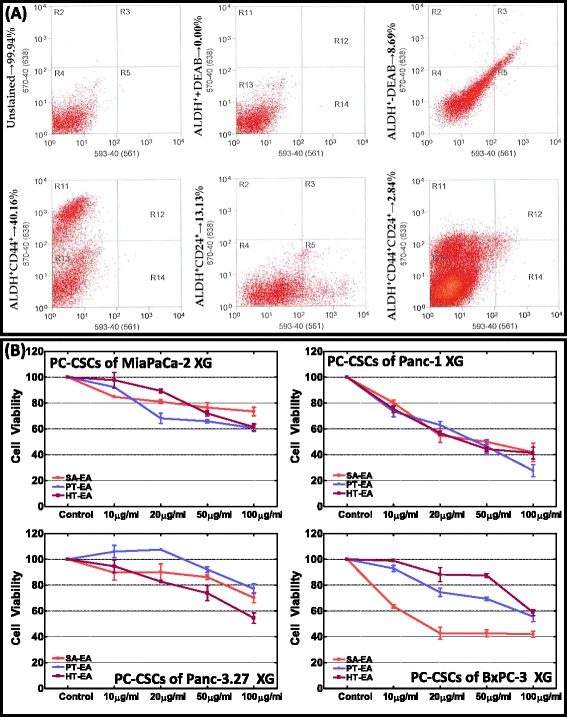
**a** Flow cytometry plots showing populations from human PC xenografts. PI^+^, mouse-CD31^+^mouse-lineage^+^, or mouse-H-2Kd^+^ cells are excluded in order to isolate only viable nonmouse-derived cells. ALDH activity was measured using ALDEFLUOR reagent (Aldefluor Kit, Stem Cell Technologies, Vancouver, BC, Canada) in the presence/absence of N,N-diethylaminobenzaldehyde (DEAB) . The CD44^+^CD24^+^ gate was created based on cells stained with ALDEFLUOR, and IgG2bk-APC (isotypic control) and IgG2ak-PE (isotypic control) antibodies. Percentage of ALDH^+^CD44^+^CD24^+^ cells is given in the text. **b** Line plots showing cell-viability dose–response curves of PC-CSCs exposed to seaweed polyphenols. PC-CSCs derived from xenografts established using MiaPACa-2, Panc-1, Panc-3.27, or BXPC-3 cells were exposed to increasing concentrations (10, 20, 50, or 100 μg/ml) of SA-EA, PT-EA, or HT-EA and examined for alterations in cell viability using the automated countess trypan blue exclusion assay. Treatment with seaweed polyphenols exhibited dose-dependent inhibition of PC-CSC cell viability with maximum inhibition at 100 μg/ml. The dose-dependent decrease in cell viability by SA-EA, HT-EA, and PT-EA remained consistent in all four PC-CSC clones investigated. *ALDH* aldehyde dehydrogenase, *CSC* cancer stem cell, *HT-EA* ethyl acetate polyphenol fraction of *Hormophysa triquerta*, *PC* pancreatic cancer, *PT-EA* ethyl acetate polyphenol fraction of *Padina tetrastromatica*, *SA-EA* ethyl acetate polyphenol fraction of *Spatoglossum asperum*

PC-CSCs from Panc-1, Panc-3.27, MiaPaCa-2, or BxPC-3 cell-derived xenografts were isolated by flow cytometry [34]. Briefly, dissociated cells were pelleted, resuspended in ALDEFLUOR buffer, and sorted by defining their phenotypes with parallel controls. We adopted the sequential exclusion → inclusion criteria to isolate human ALDH^+^CD44^+^CD24^+^ PC-CSCs from the xenograft. Cells were divided into seven groups: unstained, CD24^+^PE, CD44^+^APC, mouseCD31^−^biotin^+^mouse lineage^−^biotin^+^mouse H2Kd^−^biotin, ALDEFLUOR, ALDEFLUOR^+^DEAB^+^IgG2bκ^−^APC^+^IgG2aκ^−^PE, and ALDEFLUOR^+^CD44^+^APC^+^CD24PE^+^mouse-CD31^−^biotin^+^mouse-lineage^−^biotin^+^mouse-H-2Kd^−^biotin. CSCs were screened based on CD24^+^CD44^+^ALDH^+^ subtypes (Fig. 1a). ALDH^+^CD44^+^CD24^+^ PC-CSCs characterized and isolated from the xenografts established using MiaPaCa-2, Panc-1, Panc-3.27, and BXPC-3 were discretely collected and maintained *ex vivo* in stem cell medium (cell-line specific basal-medium without fetal bovine serum (FBS), complemented with 40 ng/ml epidermal growth factor (EGF), 10 ng/ml fibroblast growth factor (FGF), B27, N2 supplements). All sorted ALDH^+^CD44^+^CD24^+^ PC-CSCs exhibited tumorosphere formation under serum-free controlled conditions *ex vivo* (Figure S1A in Additional file 1).

### In vitro and in vivo irradiation procedures

ALDH^+^CD44^+^CD24^+^ PC-CSCs grown *ex vivo* were either mock irradiated or exposed to fractionated irradiation (FIR, 2 Gy/D for 5 days) using a Gamma Cell 40 Exactor (Best Theratronics, Ottawa, ON, Canada) at a dose rate of 0.81 Gy/minute.* In vivo* PC xenografts were selectively exposed to FIR (2 Gy/day for 5 days per week for 3 weeks) to a total dose of 30 Gy. A specially designed cerrobend shield was used to encase the bodies of the mice, exposing only the flank tumors, as described earlier [35–37]. Dosimetry was measured using both thermoluminescent and radiochromic film dosimetry [35]. Mock-irradiated animals were treated identically, except that they were not subjected to radiation.

### Polyphenol treatments

Polarity-based extractions of seaweed polyphenols were performed as described earlier [32]. For this study, ethyl acetate fractions of *Hormophysa triquerta* (HT-EA), *Spatoglossum asperum* (SA-EA), and *Padina tetrastromatica* (PT-EA) were selectively examined. Seaweeds are collected as a part of the Department of Science and Technology, Government of India-sponsored DBT project and, since this collection does not involve any endangered or protected species, no specific permissions were required (Forest Department, Government of India exempt). In brief, freshly collected, oven-dried, and powdered algae (50 g) was subjected to sequential extraction in gradient (increasing) polarity solvents including hexane, dichloromethane, ethyl acetate, and methanol for 48 h each at room temperature. The slurry in each phase was filtered and the residue was subjected for extraction in the subsequent solvent. The dried filtrates were weighed and dissolved in dimethyl sulfoxide (DMSO) to a stock concentration of 100 mg/ml. For cell treatment, stock solutions were diluted in plain cell culture medium to a “working” concentration of 10 mg/ml. For all *ex vivo *investigations, ALDH^+^CD44^+^CD24^+^ PC-CSCs were treated with 100 μg/ml of each polyphenol fraction, while a corroborated 10 mg/kg concentration was used for *in vivo* studies. The final concentration of DMSO in the cell culture medium was 0.0001 %.

### Cell viability

The trypan blue exclusion assay utilizing an automated countess was used to identify the efficacy of polyphenols in the regulation of PC-CSC cell viability. ALDH^+^CD44^+^CD24^+^ PC-CSCs (derived from xenografts established using MiaPaCa-2, Panc-1, Panc-3.27, and BxPC-3 cells) grown ex vivo were treated with 10, 20, 50, or 100 μg/ml SA-EA, PT-EA, or HT-EA, and were examined after 18 h for alterations in cell viability, as described earlier [33].

### Stem cell-related transcriptome profiling

Total RNA extraction and real-time quantitative PCR (QPCR) profiling were performed as described earlier [38, 39]. We used custom-made transcriptome profilers [40] pertaining to stemness and epithelial-to-mesenchymal transition (EMT) signaling.

### QPCR

Total RNA extraction and individual gene QPCR were performed as described earlier [41, 42]. For this study, transcriptional alterations of *Oct4*, *Zic3*, *EIF4C*, *Nanog*, and *LIF* were investigated in polyphenol-treated ALDH^+^CD44^+^CD24^+^ PC-CSCs (derived from MiaPaCa-2 and Panc-1 xenografts) exposed to FIR.

### Immunoblotting

We analyzed ALDH^+^CD44^+^CD24^+^ PC-CSCs (derived from MiaPaCa-2, Panc-1, Panc-3.27, and BXPC-3 xenografts) grown* ex vivo* and exposed to SA-EA, PT-EA, or HT-EA fractions for the alterations in Nanog, Sox2, and Oct3/4 after 24 h. Total protein extraction and immunoblotting were performed as described earlier [43].

### Tissue microarray construction and immunohistochemistry/immunofluorescence

All tissue microarray (TMA) construction procedures were performed in the SCC Cancer Tissue Pathology Core. We examined the cellular localization and expression of N-Cadherin, OCT3/4, Sox2, LIF, PI3KR1, CD44, and Nanog. Appropriate tissue morphologic/pathologic (hematoxylin and eosin (H&E)) controls and negative (no primary antibody) controls were examined in parallel. The slides were microdigitally scanned using a Scanscope and analyzed using Aperio Integrated Spectrum software (Leica Biosystems Inc., Buffalo Grove, IL, USA). For E-Cadherin, the primary protein is tagged with Mix-n-Stain™ CF™ 488A Antibody Labeling Kit (Sigma, St. Louis, MO), while the cell membrane was marked with wheat germ agglutinin (WGA)–Alexaflour-594 (Life Technologies Corporation, Grand Island, NY, USA) and nuclear counterstained with 4′,6-diamidino-2-phenylindole (DAPI). Immunofluorescence was measured in Operetta and analyzed with Columbus image data analysis.

### Oxidative stress assay

A rapid and sensitive luminescent H_2_O_2_ assay (ROS-Glo™ H_2_O_2_ Assay; Promega, Madison, WI, USA) was used to identify the efficacy of polyphenols in the regulation of PC-CSC oxidative stress. In brief, ALDH^+^CD44^+^CD24^+^ PC-CSCs (derived from MiaPaCa-2 xenograft) grown *ex vivo* were treated with 100 μg/ml SA-EA, PT-EA, or HT-EA and were allowed to incubate for 18 h. The cells were then incubated with H_2_O_2_ (25 μM final concentration) substrate solution for an additional 6 h (37 °C, 5 % CO_2_). The H_2_O_2_-proportional generated luciferin precursor was then converted to luciferin with l-cysteine and allowed to react (20 min in the dark) with Ultra Glo™ recombinant luciferase, and the luminescence was quantified using a Synergy 2 multimode reader (BioTek Instruments Inc., Winooski, VT, USA). Since polyphenols undergo abiotic reaction in cell culture medium and produce H_2_O_2_ [44, 45], appropriate “no medium”, “medium”, “medium + drug”, and “medium + cell” controls are included. All experiments were repeated three times and the background (no cells control) media factor normalized luminescence was compared between the groups using analysis of variance (ANOVA) with Bonferroni’s post-hoc comparison (GraphPad PRISM, La Jolla, CA, USA).

## Results

### Seaweed polyphenols inhibit PC-CSCs’ cell viability *ex vivo*

We analyzed MiaPaCa-2, Panc-1, Panc-3.27, and BxPC-3 cell-associated xenograft-derived PC-CSCs exposed to varying concentrations of SA-EA, PT-EA, or HT-EA for alterations in cell viability. PC-CSCs exposed to SA-EA, PT-EA, or HT-EA showed consistent inhibition of viability in concentrations as low as 10 μg/ml (Fig. 1b) in all four PC-CSC clones investigated. We observed a robust and dose-dependent inhibition of cell viability with increasing concentrations of SA-EA, PT-EA, or HT-EA with a maximum inhibition at 100 μg/ml. Panc-1-derived PC-CSCs were relatively very sensitive and demonstrated a linear dose-dependent inhibition of viability with the polyphenol treatment. All three polyphenols exerted a similar effect in this CSC clone. Other interesting observations include the following: while PT-EA exerted maximum effect in MiaPACa-2 (~40 %), Panc-1 (>60 %), and Panc-3.27 (>40 %) PC-CSCs, it exhibited relatively poor performance in Panc-3.27 PC-CSCs (>20 %); and SA-EA exerted maximum efficiency against BxPC-3 PC-CSCs (~60 %) at a concentration as low as 20 μg/ml and did not show any further increase in activity with increasing concentrations (Fig. 1b). Together, these results demonstrate that the seaweed polyphenols inflict a profound inhibition on PC-CSC cell viability.

### Seaweed polyphenols regulate the oxidative stress status of PC-CSCs

To investigate whether the exposure of seaweed polyphenols alters the oxidative stress status of the PC-CSCs, MiaPaCa-2 xenograft-derived PC-CSCs maintained ex vivo were exposed to 100 μg/ml SA-EA, PT-EA, or HT-EA and analyzed for alterations in cell-generated H_2_O_2_ utilizing the Promega cell-based ROS-Glo™ H_2_O_2_ Assay. Since polyphenols undergo abiotic reaction in cell culture medium and produce H_2_O_2_ [44, 45], appropriate in-house controls (no medium, medium, medium + drug, and medium + cell controls) are included. Compared with the untreated controls, all three polyphenols investigated exhibited a significant (*P* <0.001) regulation of oxidative stress in PC-CSCs (Figure S1B in Additional file 1). Relatively we observed a robust effect with SA-EA in the regulation of oxidative stress in PC-CSCs.

### Therapy actuates stem cell-related transcriptome in PC-CSCs

To characterize the heightened stemness of PC-CSCs that survive radiotherapy, we examined the modulations in 93 stem cell-related molecules in ALDH^+^CD44^+^CD24^+^ PC-CSCs that were derived from the xenografts established using genetically diverse MiaPaCa-2, Panc-1, Panc-3.27, and BXPC-3 cells. PC-CSCs maintained *ex vivo* were exposed to clinically relevant FIR. The FIR resulted in the robust (≥2-fold) activation of 69, 80, 74, and 77 stem cell-related molecules in PC-CSCs of MiaPaCa-2, Panc-1, Panc-3.27, and BXPC-3 origin respectively (Additional file 2). Interestingly, 43 genes (*ABCG2*, *ACAN*, *ADAR*, *ALDH1A1*, *ALDH2*, *ALPI*, *APC*, *ASCL2*, *AXIN1*, *BGLAP*, *BMP3*, *CD4*, *CD8A*, *CDC42*, *CDH1*, *COL2A1*, *COL9A1*, *CTNNA1*, *DTX1*, *DTX2*, *DVL1*, *FGFR1*, *GDF3*, *GJB1*, *GJB2*, *HDAC2*, *HRAS*, *ISL1*, *JAG1*, *MSX1*, *MYOD1*, *NOTCH2*, *NUMB*, *OCLN*, *PPARD*, *S100B*, *SHH*, *SNAI1*, *SNAI2*, *TBX2*, *TERT*, *TUBB3*, *TWIST1*) showed robust cell line-independent upregulation (Additional file 2). Further, cell lines to genes traverse analysis identified an additional 34 genes showing cell line-independent upregulation in three cell lines, and another 11 genes showing such upregulation in two cell lines. Distinctively, *GSK3B* in MiaPaCa-2-derived PC-CSCs and *NCAM1* in Panc-3.27-derived PC-CSCs showed cell line-dependent activation. Together, these data demonstrate the activation of stem cell-related molecules in PC-CSCs that survive radiation.

### Seaweed polyphenols target therapy-activated stem cell-related transcription in PC-CSCs

**Fig. 2:**
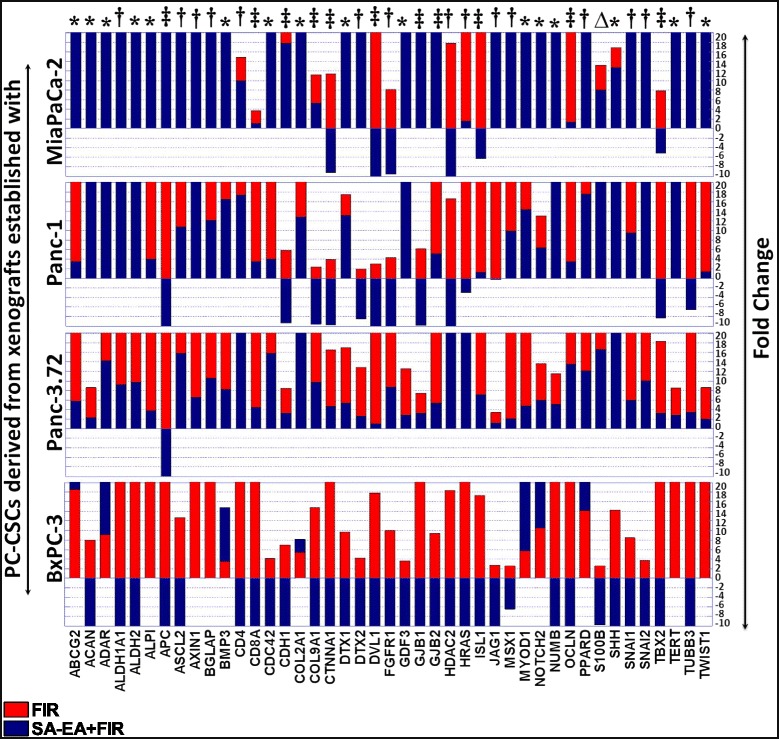
Vertical stacked bars from QPCR profiling showing the regulation of FIR-amplified stem cell-related transcription with SA-EA treatment in ALDH^+^CD44^+^CD24^+^ PC-CSCs derived from xenografts established using MiaPaCa-2, Panc-1, Panc-3.27, and BXPC-3. Inhibitory potential of SA-EA was compared for the 43 genes that showed cell line-independent increase after fractionated radiation across all four PC-CSC clones investigated. Cell line-independent effect of SA-EA for any given gene is indicated with “‡” for four, with “†” for three, and with “*” for two PC-CSC clones; “∆” for cell line-dependent effect. In the stacked bar, SA-EA + FIR bars are overlaid on top of the FIR bars and the *y* axis is restricted to a maximum of 20-fold for better presentation of comparative display. Overall, SA-EA treatment exerted cell line-independent inhibition of 11 of the 43 FIR-induced genes (*APC*, *CD8A*, *CDH1*, *COL9A1*, *CTNNA1*, *DVL1*, *GJB1*, *GJB2*, *ISL1*, *OCLN*, *TBX2*)*. CSC* cancer stem cell, *FIR* fractionated irradiation, *PC* pancreatic cancer, *SA-EA* ethyl acetate polyphenol fraction of *Spatoglossum asperum*

**Fig. 3:**
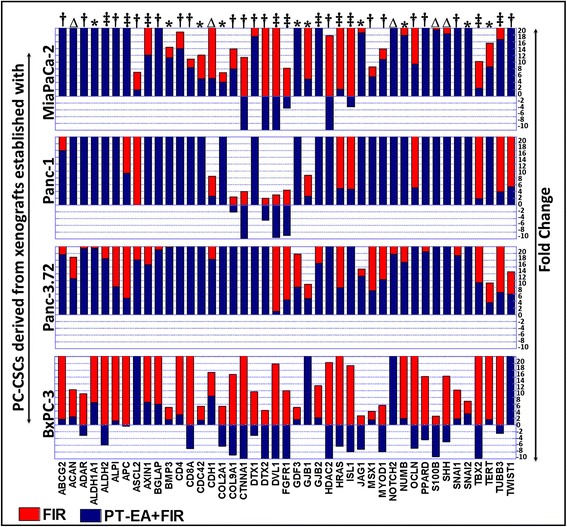
Vertical stacked bars from QPCR profiling showing the regulation of FIR-amplified stem cell-related transcription with PT-EA treatment in ALDH^+^CD44^+^CD24^+^ PC-CSCs derived from xenografts established using MiaPaCa-2, Panc-1, Panc-3.27, and BXPC-3. Inhibitory potential of PT-EA was compared for the 43 genes that showed cell line-independent increase after fractionated radiation across all four PC-CSC clones investigated. Cell line-independent effect of PT-EA for any given gene is indicated with “‡” for four, with “†” for three, and with “*” for two PC-CSC clones; “∆” for cell line-dependent effect. In the stacked bar, PT-EA + FIR bars are overlaid on top of the FIR bars and the *y* axis is restricted to a maximum of 20-fold for better presentation of comparative display. Overall, PT-EA treatment exerted cell line-independent inhibition of 10 of the 43 FIR-induced genes (*ALDH2*, *APC*, *AXIN1*, *DVL1*, *FGFR1*, *GJB2*, *HRAS*, *ISL1*, *TBX2*, *TUBB3*)*. CSC* cancer stem cell, *FIR* fractionated irradiation, *PC* pancreatic cancer, *PA-EA* ethyl acetate polyphenol fraction of *Padina tetrastromatica*

**Fig. 4:**
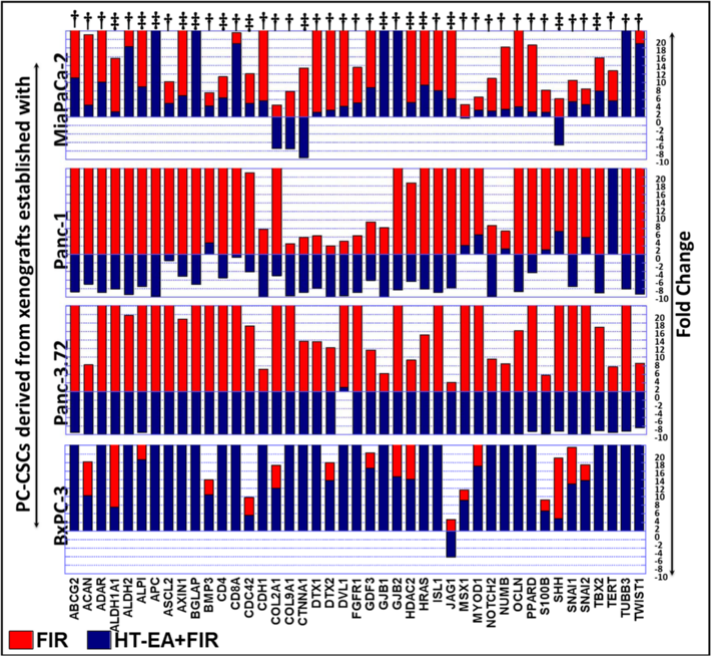
Vertical stacked bars from QPCR profiling showing the regulation of FIR-amplified stem cell-related transcription with HT-EA treatment in ALDH^+^CD44^+^CD24^+^ PC-CSCs derived from xenografts established using MiaPaCa-2, Panc-1, Panc-3.27, and BXPC-3. Inhibitory potential of HT-EA was compared for the 43 genes that showed cell line-independent increase after fractionated radiation across all four PC-CSC clones investigated. Cell line-independent effect of HT-EA for any given gene is indicated with “‡” for four, with “†” for three, and with “*” for two PC-CSC clones; “∆” for cell line-dependent effect. In the stacked bar, PT-EA + FIR bars are overlaid on top of the FIR bars and the *y* axis is restricted to a maximum of 20-fold for better presentation of comparative display. Overall, HT-EA treatment exerted cell line-independent inhibition of 14 of the 43 FIR-induced genes (*ALDH1A1*, *ALPI*, *APC*, *AXIN1*, *BGLAP*, *CD4*, *CDC42*, *CTNNA1*, *GJB1*, *HDAC2*, *HRAS*, *JAG1*, *SHH*, *TBX2*)*. CSC* cancer stem cell, *FIR* fractionated irradiation, *HT-EA* ethyl acetate polyphenol fraction of *Hormophysa triquerta*, *PC* pancreatic cancer

Next, we examined the transcriptional modulation of stem cell-related molecules in ALDH^+^CD44^+^CD24^+^ PC-CSCs pretreated with SA-EA, PT-EA, and HT-EA, and exposed to FIR. SA-EA significantly inhibited 60 (of 80), 30 (of 74), 65 (of 77), and 59 (of 69) FIR-induced (≥2-fold) stem cell-related molecules in Panc-1, MiaPaCa-2, Panc-3.27, and BXPC-3-derived PC-CSCs respectively. While 11 of the 43 cell line-independent FIR-induced genes (*APC*, *CD8A*, *CDH1*, *COL9A1*, *CTNNA1*, *DVL1*, *GJB1*, *GJB2*, *ISL1*, *OCLN*, *TBX2*) were completely suppressed by SA-EA treatment across all four PC-CSC clones investigated (Fig. 2‡), we also observed a cell line-independent inhibition of 15 genes (*ALDH1A1*, *ASCL2*, *AXIN1*, *BGLAP*, *CD4*, *DTX2*, *FGFR1*, *HDAC2*, *HRAS*, *JAG1*, *MSX1*, *PPARD*, *SNAI1*, *SNAI2*, *TUBB3*) in at least three PC-CSC clones (Fig. 2†) and 16 other genes (*ABCG2*, *ACAN*, *ADAR*, *ALDH2*, *ALPI*, *BMP3*, *CDC42*, *COL2A1*, *DTX1*, *GDF3*, *MYOD1*, *NOTCH2*, *NUMB*, *SHH*, *TERT*, *TWIST1*) in two PC-CSC clones (Fig. 2*). However, cell line-dependent inhibition of *S100β* in PC-CSCs of BXPC-3 origin with SA-EA was also observed. Likewise, PT-EA treatment completely inhibited 46, 37, 43, and 65 of 80, 74, 77, and 69 FIR-induced stem cell-related molecules in Panc-1, MiaPaCa-2, Panc-3.27, and BXPC-3-derived PC-CSCs. However, of 43 cell line-independent FIR-induced genes, the comparison analysis revealed a cell line-independent inhibition of 10 genes (*ALDH2*, *APC*, *AXIN1*, *DVL1*, *FGFR1*, *GJB2*, *HRAS*, *ISL1*, *TBX2*, *TUBB3*) in all four PC-CSC clones (Fig. 3‡). In addition, a set of 17 genes (*ABCG2*, *ADAR*, *ALPI*, *ASCL2*, *BGLAP*, *CD4*, *CD8A*, *COL9A1*, *CTNNA1*, *DTX1*, *DTX2*, *MSX1*, *MYOD1*, *OCLN*, *PPARD*, *SNAI1*, *TWIST1*) inhibited in common in at least three PC-CSC clones (Fig. 3†), while another 11 genes (*ALDH1A1*, *BMP3*, *CDC42*, *COL2A1*, *GDF3*, *GJB1*, *HDAC2*, *JAG1*, *NUMB*, *SNAI2*, *TERT*) were completely inhibited in two PC-CSCs with PT-EA (Fig. 3*). However, PT-EA silenced a small subset of genes (*ACAN*, *S100β*, *SHH* in BXPC-3; *CDH1* in MiaPaCa-2; *NOTCH2* in Panc-1 PC-CSC) in a cell line-dependent manner*.* Pretreating cells with HT-EA inhibited 80 (of 80), 72 (of 74), 75 (of 77), and 23 (of 69) FIR-induced stem cell-related molecules in Panc-1, MiaPaCa-2, Panc-3.27, and BXPC-3-derived ALDH^+^CD44^+^CD24^+^ PC-CSCs. Interestingly, 14 of 43 FIR-induced genes (*ALDH1A1*, *ALPI*, *APC*, *AXIN1*, *BGLAP*, *CD4*, *CDC42*, *CTNNA1*, *GJB1*, *HDAC2*, *HRAS*, *JAG1*, *SHH*, *TBX2*) were completely suppressed with HT-EA treatment in all four PC-CSCs investigated (Fig. 4‡). We observed a significant cell line-independent inhibition of 29 (*ABCG2*, *ACAN*, *ADAR*, *ALDH2*, *ASCL2*, *BMP3*, *CD8A*, *CDH1*, *COL2A1*, *COL9A1*, *DTX1*, *DTX2*, *DVL1*, *FGFR1*, *GDF3*, *GJB2*, *ISL1*, *MSX1*, *MYOD1*, *NOTCH2*, *NUMB*, *OCLN*, *PPARD*, *S100B*, *SNAI1*, *SNAI2*, *TERT*, *TUBB3*, *TWIST1*) in three PC-CSCs with HT-EA treatment (Fig. 4†). Evidently, FIR-induced *APC* and *TBX2* were completely and consistently inhibited by all three seaweed polyphenols tested in all four PC-CSC clones investigated. Together, these data demonstrate the selective FIR-induced stem cell-related transcriptome inhibitory potential of SA-EA, PT-EA, and HT-EA in PC-CSCs that survive radiotherapy.

### Seaweed polyphenols target radiotherapy-activated EIF4C, OCT3/4, Nanog, LIF, and ZIC3 in PC-CSCs

**Fig. 5:**
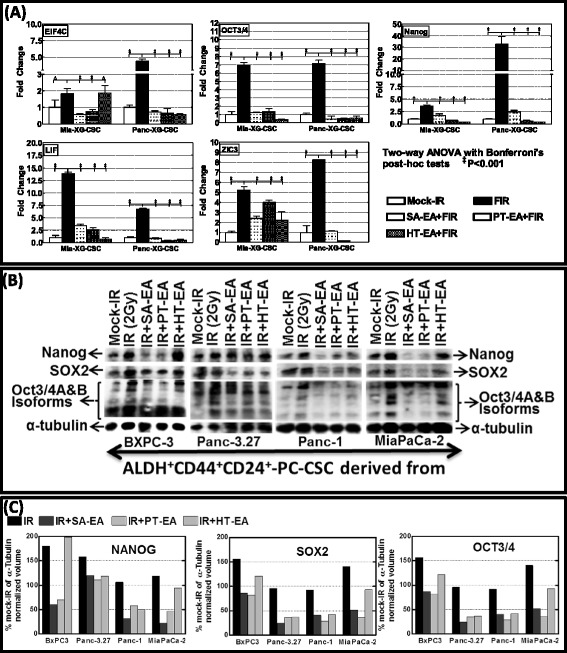
**a** QPCR analysis showing mRNA levels of *EIF4C*, *OCT3/4*, *Nanog*, *LIF*, and *ZIC3* in ALDH^+^CD44^+^CD24^+^ PC-CSCs derived from xenografts of Panc-1 and MiaPaCa-2, either mock irradiated or exposed to FIR, with or without 100 μg/ml SA-EA, PT-EA, or HT-EA. **b** Representative immunoblots showing expression levels of Nanog, Sox2, and Oct3/4 in Panc-1, MiaPaCa-2, Panc-3.27, and BXPC-3-derived ALDH^+^CD44^+^CD24^+^ PC-CSCs, either mock irradiated or exposed to radiation, with or without SA-EA, PT-EA, or HT-EA. **c** Histograms show the Quantity-One densitometry of relative band intensities. *ALDH* aldehyde dehydrogenase, *ANOVA* analysis of variance, *CSC* cancer stem cell, *FIR* fractionated irradiation, *HT-EA* ethyl acetate polyphenol fraction of *Hormophysa triquerta*, *PC* pancreatic cancer, *PT-EA* ethyl acetate polyphenol fraction of *Padina tetrastromatica*, *SA-EA* ethyl acetate polyphenol fraction of *Spatoglossum asperum*

To further recognize the regulation potential of seaweed polyphenols in the stem cell-related transcriptional machinery that drives EMT, pluripotency maintenance, and the self-renewal capacity of PC-CSCs, we examined the transcriptional modulations of *EIF4C*, *OCT3/4*, *Nanog*, *LIF*, and *ZIC3* in PC-CSCs. *SOX2*, *OCT3/4*, *Nanog*, *LIF*, *EIF4C*, and *ZIC3* comprise the core regulatory circuitry in stem cells that overturns differentiation-associated genes, and determines the improved stemness, pluripotency maintenance, and self-renewal capacity. Since these critical players (except for *Sox2*) are not included in the QPCR profiling, we investigated whether radiation activates these stemness-maintenance markers in PC-CSCs and further defined the benefit of seaweed polyphenols in mitigating such transcriptional/translational response. First, we examined the benefit of seaweed polyphenols in targeting *EIF4C*, *OCT3/4*, *Nanog*, *LIF*, and *ZIC3* transcriptional activation as a standalone drug. For this, *ex vivo* maintained PC-CSCs (derived using MiaPaCa-2 and Panc-1 cells) were exposed to 100 μg/ml SA-EA, PT-EA, or HT-EA and examined after 24 h. Overall, SA-EA, PT-EA, or HT-EA treatment exhibited a significant and consistent transcriptional inhibition of *EIF4C*, *OCT3/4*, *Nanog*, *LIF*, and *ZIC3* in both MiaPaCa-2 and Panc-1-derived PC-CSCs (Figure S1C in Additional file 1). Evidently, SA-EA and PT-EA treatment exerted significant inhibition of *OCT3/4*, *Nanog*, and *LIF* in both PC-CSCs investigated. On the other hand, exposing the cells to HT-EA resulted in the complete inhibition of *OCT3/4* in both PC-CSCs. Next, we examined the transcriptional modulation of *EIF4C*, *OCT3/4*, *Nanog*, *LIF*, and *ZIC3* in MiaPaCa-2 and Panc-1-derived ALDH^+^CD44^+^CD24^+^ PC-CSCs pretreated with SA-EA, PT-EA, and HT-EA, and exposed to radiotherapy. Therapy-associated activation of *EIF4C* in both PC-CSC clones was significantly inhibited in the presence of SA-EA, PT-EA, and HT-EA (Fig. 5a). Consistently, all three fractions inflict cell line-independent inhibition of FIR-induced *OCT3/4* transcription in PC-CSCs (Fig. 5a). Moreover, we observed significant inhibition of *Nanog* transactivation in both PC-CSCs with SA-EA, PT-EA, and HT-EA (Fig. 5a). Further, QPCR analysis recognized the definite inhibition of radiotherapy-induced *LIF* transactivation in PC-CSCs in the presence of seaweed polyphenols (Fig. 5a). Zic3 that was significantly activated in response to therapy was completely suppressed in PC-CSCs with SA-EA, PT-EA, or HT-EA (Fig. 5a). Immunoblotting revealed that the SA-EA, PT-EA, or HT-EA profoundly regulate therapy-induced expression of pluripotency, maintaining Nanog, SOX2, and OCT3/4 in MiaPaCa-2, Panc-1, Panc-3.27, and BXPC-3-derived ALDH^+^CD44^+^CD24^+^ PC-CSCs (Fig. 5b). Band intensity densitometry results corroborated well with the transcription data and recognized the potential of these drug deliverables in stemness-related functional response (Fig. 5c).

### Seaweed polyphenols regulate SOX2, PI3KR1, OCT3/4, N-Cadherin, E-Cadherin, Nanog, LIF, and CD44 in residual PC *in vivo*

**Fig. 6:**
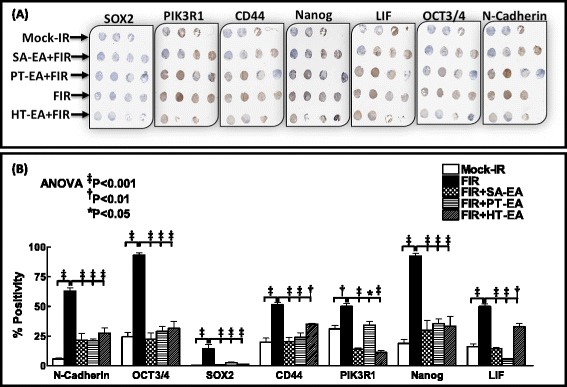
**a** TMAs labeled for SOX2, PIK3R1, CD44, Nanog, LIF, OCT3/4, and N-Cadherin. **b** Aperio-TMA quantitation analysis showing protein-specific positivity magnitudes in mock-irradiated xenografts and residual PC tissues after radiotherapy, with or without SA-EA, PT-EA, or HT-EA. *ANOVA* analysis of variance, *FIR* fractionated irradiation, *HT-EA* ethyl acetate polyphenol fraction of *Hormophysa triquerta*, *PT-EA* ethyl acetate polyphenol fraction of *Padina tetrastromatica*, *SA-EA* ethyl acetate polyphenol fraction of *Spatoglossum asperum*

To further substantiate our *ex vivo* findings, we examined the cellular expression/localization levels of SOX2, PI3KR1, OCT3/4, N-Cadherin, E-Cadherin, Nanog, LIF, and CD44 in residual PC. SOX2 staining revealed basal levels (0.31 ± 0.13 %) of positivity in mock-irradiated xenografts (Fig. 6). The positivity of SOX2 appeared in dark brown, and was principally localized in the nucleus (pullout in Additional file 3). SOX2 immunoreactivity was intense (14.35 ± 3.68 %) in residual tumors after FIR (Fig. 6; Additional file 3). We observed a complete loss of FIR-induced SOX2 with SA-EA, PT-EA, or HT-EA (Fig. 6b). Expression of PIK3R1 was stronger in tumor cells than in stromal cells, and was predominantly localized in plasma membranes as well as in the cytoplasm (pullout in Additional file 4). Radiotherapy-treated xenografts demonstrated strong (49.98 ± 2.38 %) PIK3R1 expression (Fig. 6b; Additional file 4). Although SA-EA and PT-EA showed low levels of PIK3R1 compared with the FIR group, PIK3R1 was barely detectable with HT-EA treatment. OCT3/4-stained nuclei appeared in dark brown and were found mainly in epithelial cells (pullout in Additional file 5). OCT3/4 positivity ranged from 24.36 ± 3.7 % in mock-irradiated cells to 93.29 ± 1.96 % of irradiated residual tumors (Fig. 6b; Additional file 5). The range for OCT3/4-positive epithelial cells was 16–38 % (SA-EA), <30 % (PT-EA), and 31.55 ± 5.85 % (HT-EA) with seaweed polyphenol treatment.

Positive staining of N-Cadherin appeared in dark brown and was predominantly localized in cell membranes (pullout in Additional file 6). Compared with mock-IR, N-Cadherin immunoreactivity was intense in residual tumors after FIR (Fig. 6b; Additional file 6). We observed a significant (*P* <0.001) reduction in N-Cadherin immunoreactivity in the tumors of mice that received SA-EA, PT-EA, and HT-EA. Likewise, radiotherapy exerted intense Nanog immunoreactivity that was predominantly localized in the nucleus (pullout in Additional file 7). SA-EA, PT-EA, and HT-EA pretreatment showed weak nuclear Nanog (Fig. 6b; Additional file 7). LIF-positive staining appeared in brown, and was localized in plasma membranes, the cytoplasm, and nuclei (pullout in Additional file 8). However, radiotherapy-treated cells exhibited high LIF immunoreactivity. Regulation of the radiotherapy-induced LIF was evident in PC tissues of animals that received SA-EA, PT-EA, and HT-EA (Fig. 6b; Additional file 8). CD44 immunoreactivity was highly intense and present in >50 % of the PC cells in residual tumors after FIR (Fig. 6b). The positivity of CD44 appeared in dark brown, and was predominantly localized in plasma membranes and the cytoplasm (pullout in Additional file 9 for granular cytoplasmic expression). Conversely, SA-EA, PT-EA, and HT-EA treatment exerted significant regulation of FIR-induced CD44 in residual PC tumors (Additional file 9). We observed a strong E-Cadherin immunoreactivity in mock-irradiated tumors. The positivity of E-Cadherin appeared in green, and was predominantly localized in plasma membranes and the cytoplasm (pullout in Additional file 10). Immunofluorescence was barely detectable (<20 %) in the PC cells in residual tumors after clinical doses of FIR (Additional file 10). Interestingly, treatment with SA-EA, PT-EA, and HT-EA following radiotherapy resulted on a profound increase in E-Cadherin localization in residual PC tumors.

## Discussion

The results of this study demonstrated the survival and transcriptional activation of stem cell-related molecules in ALDH^+^CD44^+^CD24^+^ PC-CSCs after first-line therapy. For the first time, the results presented here defined the clinical potential of seaweed polyphenols in definite, comprehensive inhibition of stemness-related molecular turmoil in PC-CSCs surviving after therapy. Considering the magnitude of acquired (or via clonal selection) EMT/CSC physiognomies in PC cells that survive a course of therapy, the current study provides a molecular blueprint to underscore the importance of CSC targeting for a better PC cure. Although a number of studies have focused on identifying the role of PC-CSCs in drug resistance [46, 47], the selection of CSC clones and/or acquired EMT-CSC phenotypes in response to therapy and their functional roles in PC relapse (or for that matter in any tumors) are poorly understood. To our knowledge, this study was the first of its kind portraying the amplified EMT/CSC-related molecular physiognomies after first-line therapy in PC. Moreover, this study employs both an *in vivo* preclinical approach and an *ex vivo* bench approach, and demonstrates the intensified stemness in the cells surviving after therapy. The results exposed highly comparable activation of stemness machinery between every PC-CSC clone, depicting a similar therapy-associated acquisition and/or enrichment of EMT/stem characteristics, regardless of the cell of origin.

Seaweed polyphenol (SA-EA, PT-EA, or HT-EA) treatment exerted a complete suppression of therapy-induced Nanog, Oct-4, and Sox-2 in PC-CSCs. These pluripotent CSCs maintaining Nanog, Oct-4, and Sox-2 exhibit tight interaction and regulate their own promoters, and along with other key developmental genes comprehensively form autoregulatory loops and functional signaling [48]. We observed significantly high levels of Nanog in therapy-resistant PC-CSCs ex vivo as well as in residual PC. Evidently, a high level of Nanog is a key regulator of stem cell self-renewal, pluripotency, and clonal expansion [49, 50] and it has been shown that Nanog-deficient cells lose their pluripotency [49]. Voluminous evidence implicates Nanog, Sox2, and OCT-4 in the stemness and pathogenesis of PC [51–53]. In the present study, the inhibition of therapy activated Nanog, SOX2, and OCT-4 by the seaweed polyphenol treatment, suggesting that comprehensive inhibition of these self-orchestrating CSC pluripotency maintaining factors could be a novel strategy to prevent stemness-maintenance in therapy-resistant PC-CSCs.

In addition, the results of the present study portray for the first time the significant inhibition of therapy-orchestrated N-Cadherin by the seaweed polyphenols. EMT, an event in which epithelial cells drop their characteristics and gain mesenchymal features, plays a crucial role in tumor progression. To that end, tumor metastasis requires the dissemination of CSCs from the primary site and subsequently colonizes in a distant site. In this regard, EMT has been shown to actively instigate stromal invasion, intravasation, dissemination, and colonization at distant sites, all crucial and mandatory events of metastasis. Notably, studies have shown that EMT predicts prognosis and contributes to the drug resistance in PC [54, 55], and a number of EMT markers are recognized in PC [56]. Consistently, results of our study show a robust increase in the cellular levels of N-Cadherin in residual PC after clinical doses of radiotherapy. Since its first implication for EMT in PC [57], several studies documented its importance and signaling in PC progression, and focused on identifying drug deliverables against N-Cadherin [58–60]. However, thus far drug deliverables targeting EMT of drug/therapy resistant cells are still unexplored. To our knowledge, for the first time, the results of the present study demonstrate that the seaweed polyphenols inhibit the expression of N-Cadherin in therapy-resistant residual tumors and further imply that these fractions could effectively target EMT-driven drug/therapy resistance in PC.

CSCs have been shown to play critical roles in drug resistance and tumor metastasis in many human malignancies including PC. It is now evident that PC-CSCs are resistant to well-defined chemotherapy and radiotherapy, and contribute to PC metastasis and recurrence after treatment by regenerating multiple cell types in the tumor through their maintained stemness. Since it is clear that the therapeutic failure/recurrence is due to ineffective targeting of the PC-CSC population, drug deliverables that selectively target PC-CSCs offer a greater promise for PC cure as well as prevention of recurrence. In this regard, the results of the present study defined the stemness-related molecular blue print in therapy-resistant PC-CSCs derived from primary tumors established with genetically diverse human PC cells. Moreover, this study identified the preclinical efficacy of seaweed polyphenols in targeting PC-CSC viability and therapy-associated activation of PC-CSC pluripotency and stemness-maintenance. The outcome of this study recognizes the potential benefits of these seaweed polyphenols as adjuvants for the current PC therapeutic modalities in place, but further studies with appropriate transgenic mouse models of spontaneous pancreatic carcinogenesis are warranted.

## Conclusions

Remarkably, we identified significant benefits of the select high-polarity antioxidant-rich seaweed polyphenols in all-inclusive targeting of the intensified stemness in residual PC cells. The results defined the clinical efficacy of these polyphenols by demonstrating clone-independent inhibition of stem cell-related molecules. Since current conventional chemotherapy and radiotherapy are largely ineffective in depleting CSCs, identifying novel therapeutic strategies to selectively target CSCs is of utmost importance. Since aberrant reactivation of multiple signaling pathways is involved in the formation of PC-CSCs, identifying drugs that could target these events could be considered highly beneficial. Although many studies have focused on identifying the benefits of polyphenols from various sources to target the stem cell self-renewal capacity and pluripotency maintenance, these studies focused only on specific signaling events and understanding PC-CSCs before therapy. For the first time, this study identified three seaweed polyphenol extractions that possess proven anti-PC potential, and the ability to target the main signaling pathways that play critical roles in activating and/or maintaining CSCs after first-line therapy. In this regard, the results presented in this study are the front-runner for understanding the potential of polyphenols in therapy-orchestrated activation of PC-CSCs. However, further studies are needed to characterize these polyphenols in the perspective of pharmacokinetics, normal tissue cytotoxicity, in vivo bioavailability, and drug-delivering moieties to identify potential drug deliverables that could generate an archetypal shift in PC treatment practices.

## Additional files

Additional file 1: Figure S1.
**A** Representative photomicrographs showing the tumorosphere-forming capacity of ALDH^+^CD44^+^CD24^+^ PC-CSCs. MiaPaCa-2 xenograft-derived PC-CSCs maintained ex vivo in serum free stem-cell culture conditions showing the cellular aggregation pattern and formation of organized tumorosphere. **B** Histograms obtained from rapid and sensitive luminescent ROS-Glo™ H_2_O_2_ Assay showing significant regulation of oxidative stress status in ALDH^+^CD44^+^CD24^+^ PC-CSCs (derived from MiaPaCa-2 xenograft) exposed to SA-EA, PT-EA, or HT-EA. Group-wise comparisons were made by ANOVA with Tukey’s post-hoc correction using GraphPad PRISM. **C** Histograms of QPCR analysis showing mRNA levels of EIF4C, OCT3/4, Nanog, LIF, and ZIC3 in ALDH^+^CD44^+^CD24^+^ PC-CSCs derived from xenografts of Panc-1 and MiaPaCa-2 exposed with or without 100 μg/ml SA-EA, PT-EA, or HT-EA. Overall, seaweed polyphenols as a standalone compound significantly reduced the transcriptional activation of inhibited EIF4C, OCT3/4, Nanog, LIF, and ZIC3 in PC-CSCs. (PDF 1511 kb) Additional file 2: Figure S2. Showing clinical doses of radiation (2 Gy/day for 5 days, for a total dose of 10 Gy) significantly induced (≥2-fold upregulation) EMT and stem cell-related transcriptome in PC-CSCs derived from Panc-1, Panc-3.27, MiaPaCa-2, or BxPC-3 cells’ established xenografts. Forty-three genes (*ABCG2*, *ACAN*, *ADAR*, *ALDH1A1*, *ALDH2*, *ALPI*, *APC*, *ASCL2*, *AXIN1*, *BGLAP*, *BMP3*, *CD4*, *CD8A*, *CDC42*, *CDH1*, *COL2A1*, *COL9A1*, *CTNNA1*, *DTX1*, *DTX2*, *DVL1*, *FGFR1*, *GDF3*, *GJB1*, *GJB2*, *HDAC2*, *HRAS*, *ISL1*, *JAG1*, *MSX1*, *MYOD1*, *NOTCH2*, *NUMB*, *OCLN*, *PPARD*, *S100B*, *SHH*, *SNAI1*, *SNAI2*, *TBX2*, *TERT*, *TUBB3*, *TWIST1*) showed consistent cell line-independent upregulation. (PDF 839 kb) Additional file 3: Figure S3. Showing representative microphotographs from SOX2-stained PC TMA constructed with xenografts (established from MiaPaCa-2) exposed to mock irradiation or fractionated irradiation, with or without SA-EA, PT-EA, and HT-EA fractions. Pullout shows the staining pattern (20× magnification). (PDF 2283 kb) Additional file 4: Figure S4. Showing representative microphotographs from PIK3R1-stained PC TMA constructed with xenografts (established from MiaPaCa-2) exposed to mock irradiation or fractionated irradiation, with or without SA-EA, PT-EA, and HT-EA fractions. Pullout shows the staining pattern (20× magnification). (PDF 2998 kb) Additional file 5: Figure S5. Showing representative microphotographs from OCT3/4-stained PC TMA constructed with xenografts (established from MiaPaCa-2) exposed to mock irradiation or fractionated irradiation, with or without SA-EA, PT-EA, and HT-EA fractions. Pullout shows the staining pattern (20× magnification). (PDF 2892 kb) Additional file 6: Figure S6. Showing representative microphotographs from N-Cadherin-stained PC TMA constructed with xenografts (established from MiaPaCa-2) exposed to mock irradiation or fractionated irradiation, with or without SA-EA, PT-EA, and HT-EA fractions. Pullout shows the staining pattern (20× magnification). (PDF 3032 kb) Additional file 7: Figure S7. Showing representative microphotographs from Nanog-stained PC TMA constructed with xenografts (established from MiaPaCa-2) exposed to mock irradiation or fractionated irradiation, with or without SA-EA, PT-EA, and HT-EA fractions. Pullout shows the staining pattern (20× magnification). (PDF 4861 kb) Additional file 8: Figure S8. Showing representative microphotographs from LIF-stained PC TMA constructed with xenografts (established from MiaPaCa-2) exposed to mock irradiation or fractionated irradiation, with or without SA-EA, PT-EA, and HT-EA fractions. Pullout shows the staining pattern (20× magnification). (PDF 2688 kb) Additional file 9: Figure S9. Showing representative microphotographs from CD44-stained PC TMA constructed with xenografts (established from MiaPaCa-2) exposed to mock irradiation or fractionated irradiation, with or without SA-EA, PT-EA, and HT-EA fractions. Pullout shows the staining pattern (20× magnification). (PDF 2910 kb) Additional file 10: Figure S10. Showing representative microphotographs from E-Cadherin-stained PC TMA constructed with xenografts (established from MiaPaCa-2) exposed to mock-irradiation or fractionated irradiation, with or without SA-EA, PT-EA, and HT-EA fractions. Pullout shows the staining pattern (20× magnification). (PDF 2643 kb)

## Abbreviations

ALDH: Aldehyde dehydrogenase
ANOVA: Analysis of variance
CSC: Cancer stem cell
DAPI: 4′,6-Diamidino-2-phenylindole
DMSO: Dimethyl sulfoxide
EGF: Epidermal growth factor
EMT: Epithelial-to-mesenchymal transition
FBS: Fetal bovine serum
FGF: Fibroblast growth factor
FIR: Fractionated irradiation
Gy: Gray, a derived unit of ionizing radiation
H&E: Hematoxylin and eosin
HT-EA: Ethyl acetate polyphenol fraction of *Hormophysa triquerta*
PC: Pancreatic cancer
PT-EA: Ethyl acetate polyphenol fraction of *Padina tetrastromatica*
QPCR: Quantitative PCR
SA-EA: Ethyl acetate polyphenol fraction of *Spatoglossum asperum*
TMA: Tissue microarray
WGA: Wheat germ agglutinin
