# Supplementary figures and images for: Polyphenols from marine brown algae target radiotherapy-coordinated EMT and stemness-maintenance in residual pancreatic cancer
Source: Stem Cell Res Ther. 2015 Sep 22;6(1):182. doi: 10.1186/s13287-015-0173-3 (PMC4578749; doi:10.1186/s13287-015-0173-3)

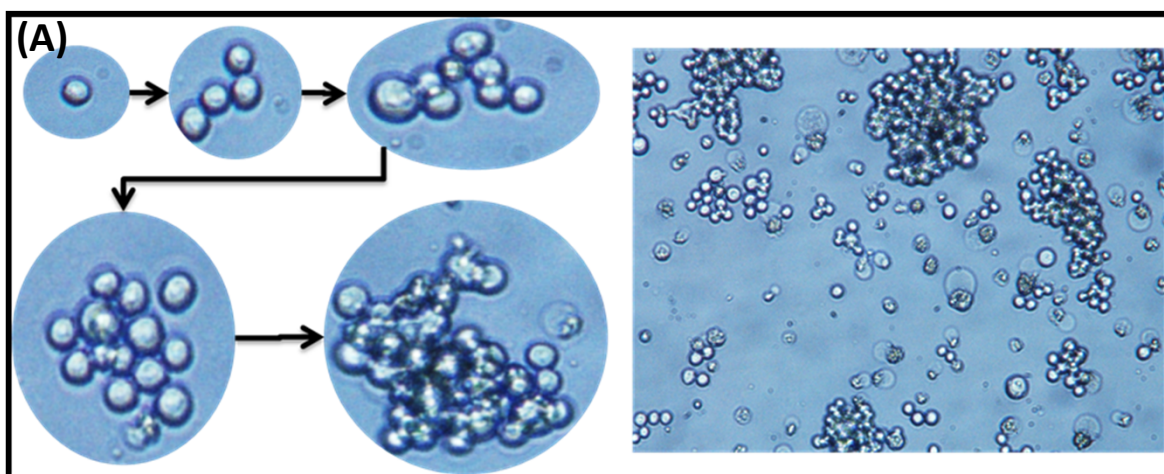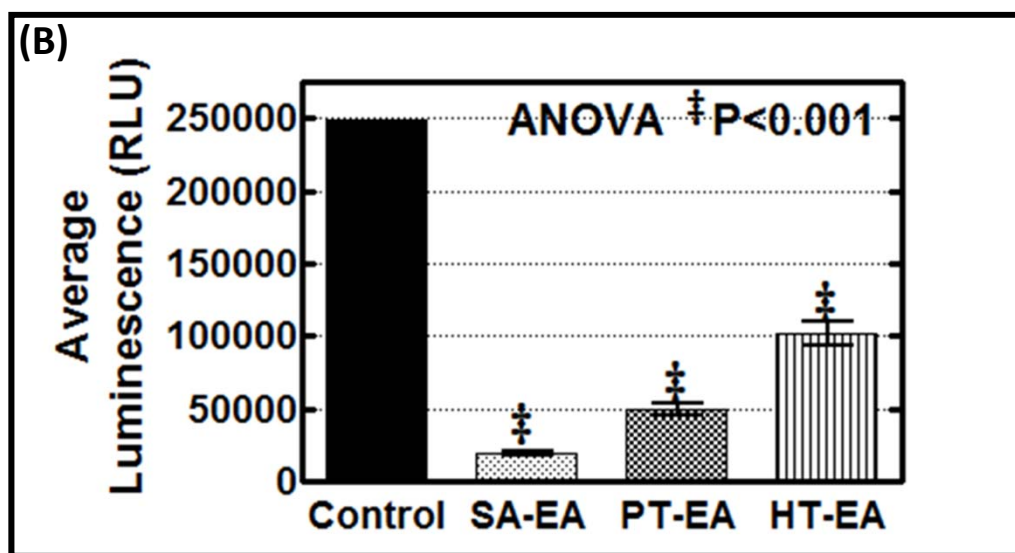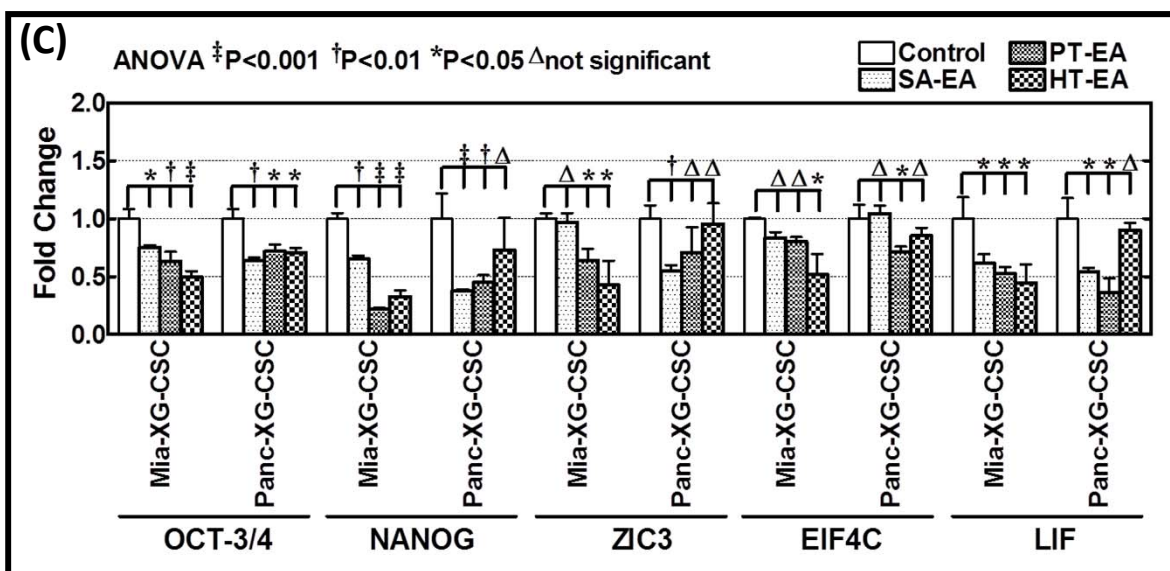

Supplement: Additional file 1: Figure S1. — A Representative photomicrographs showing the tumorosphere-forming capacity of ALDH+CD44+CD24+ PC-CSCs. MiaPaCa-2 xenograft-derived PC-CSCs maintained ex vivo in serum free stem-cell culture conditions showing the cellular aggregation pattern and formation of organized tumorosphere. B Histograms obtained from rapid and sensitive luminescent ROS-Glo™ H2O2 Assay showing significant regulation of oxidative stress status in ALDH+CD44+CD24+ PC-CSCs (derived from MiaPaCa-2 xenograft) exposed to SA-EA, PT-EA, or HT-EA. Group-wise comparisons were made by ANOVA with Tukey’s post-hoc correction using GraphPad PRISM. C Histograms of QPCR analysis showing mRNA levels of EIF4C, OCT3/4, Nanog, LIF, and ZIC3 in ALDH+CD44+CD24+ PC-CSCs derived from xenografts of Panc-1 and MiaPaCa-2 exposed with or without 100 μg/ml SA-EA, PT-EA, or HT-EA. Overall, seaweed polyphenols as a standalone compound significantly reduced the transcriptional activation of inhibited EIF4C, OCT3/4, Nanog, LIF, and ZIC3 in PC-CSCs. (PDF 1511 kb) [file 13287_2015_173_MOESM1_ESM.pdf]

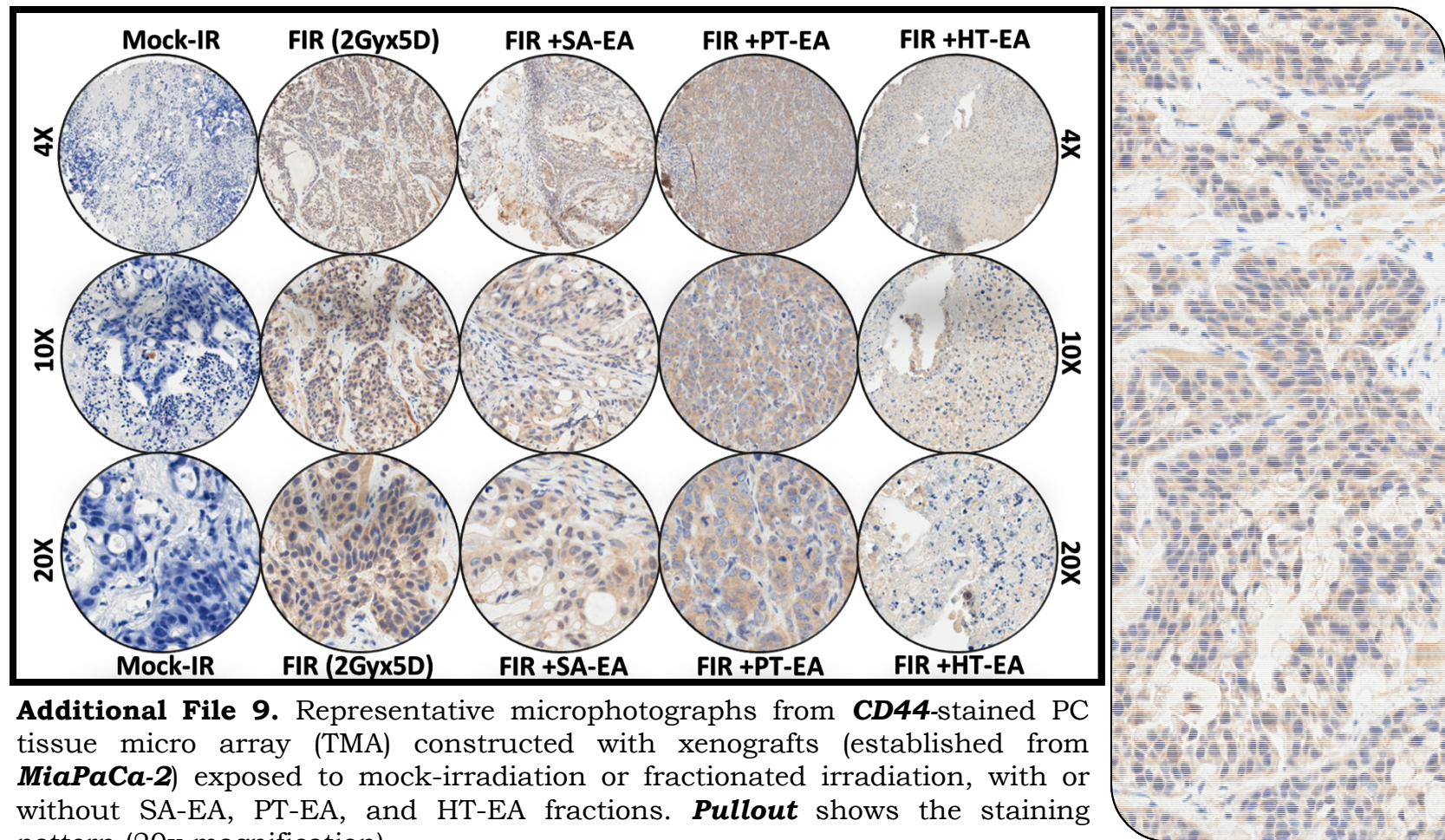

Supplement: Additional file 9: Figure S9. — Showing representative microphotographs from CD44-stained PC TMA constructed with xenografts (established from MiaPaCa-2) exposed to mock irradiation or fractionated irradiation, with or without SA-EA, PT-EA, and HT-EA fractions. Pullout shows the staining pattern (20× magnification). (PDF 2910 kb) [file 13287_2015_173_MOESM9_ESM.pdf]

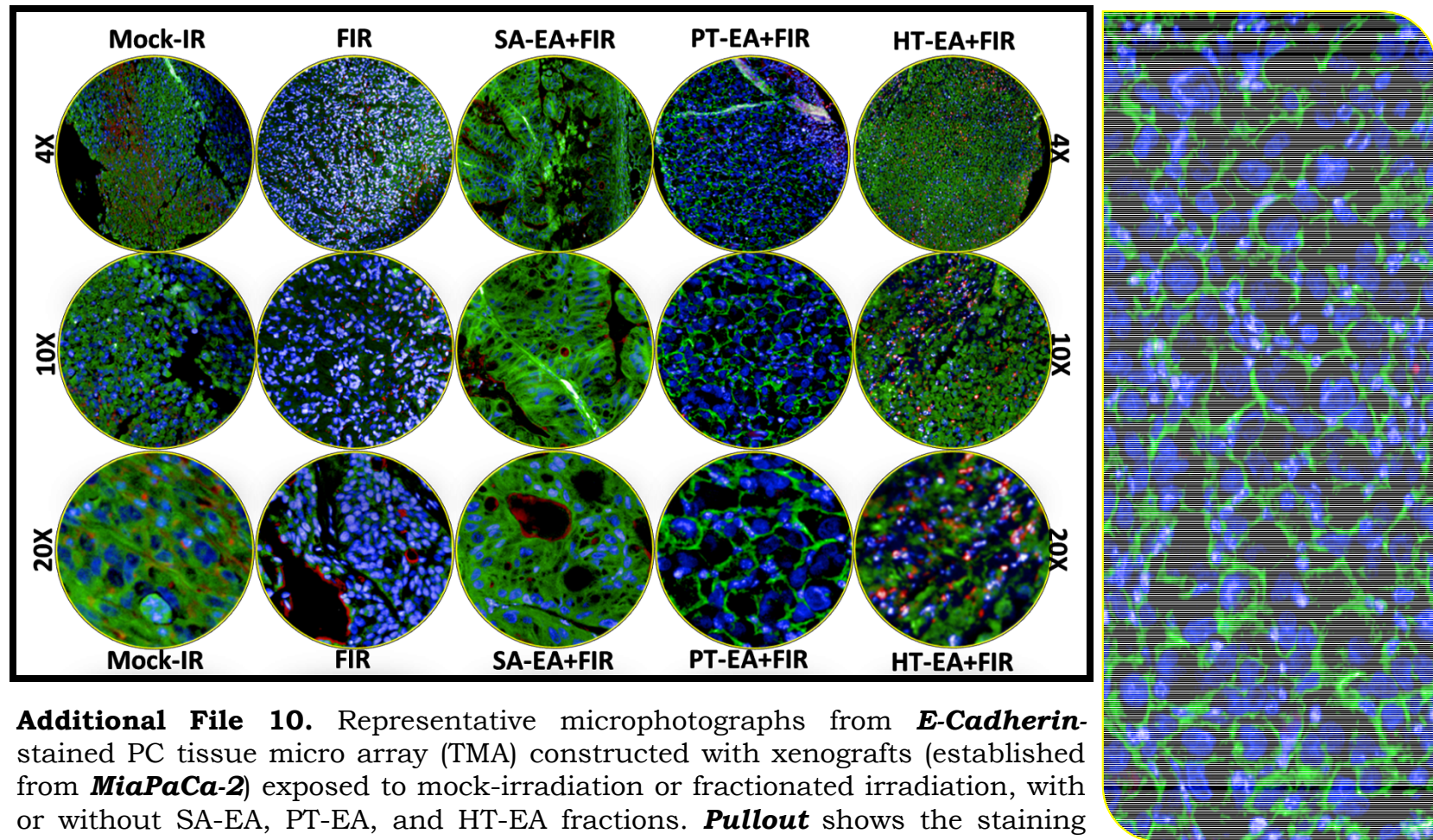

Supplement: Additional file 10: Figure S10. — Showing representative microphotographs from E-Cadherin-stained PC TMA constructed with xenografts (established from MiaPaCa-2) exposed to mock-irradiation or fractionated irradiation, with or without SA-EA, PT-EA, and HT-EA fractions. Pullout shows the staining pattern (20× magnification). (PDF 2643 kb) [file 13287_2015_173_MOESM10_ESM.pdf]
