# Supplementary material for: Polyphenols from marine brown algae target radiotherapy-coordinated EMT and stemness-maintenance in residual pancreatic cancer
Source: Stem Cell Res Ther. 2015 Sep 22;6(1):182. doi: 10.1186/s13287-015-0173-3 (PMC4578749; doi:10.1186/s13287-015-0173-3)
Supplement: Additional file 2: Figure S2. — Showing clinical doses of radiation (2 Gy/day for 5 days, for a total dose of 10 Gy) significantly induced (≥2-fold upregulation) EMT and stem cell-related transcriptome in PC-CSCs derived from Panc-1, Panc-3.27, MiaPaCa-2, or BxPC-3 cells’ established xenografts. Forty-three genes (ABCG2, ACAN, ADAR, ALDH1A1, ALDH2, ALPI, APC, ASCL2, AXIN1, BGLAP, BMP3, CD4, CD8A, CDC42, CDH1, COL2A1, COL9A1, CTNNA1, DTX1, DTX2, DVL1, FGFR1, GDF3, GJB1, GJB2, HDAC2, HRAS, ISL1, JAG1, MSX1, MYOD1, NOTCH2, NUMB, OCLN, PPARD, S100B, SHH, SNAI1, SNAI2, TBX2, TERT, TUBB3, TWIST1) showed consistent cell line-independent upregulation. (PDF 839 kb) [file 13287_2015_173_MOESM2_ESM.pdf]

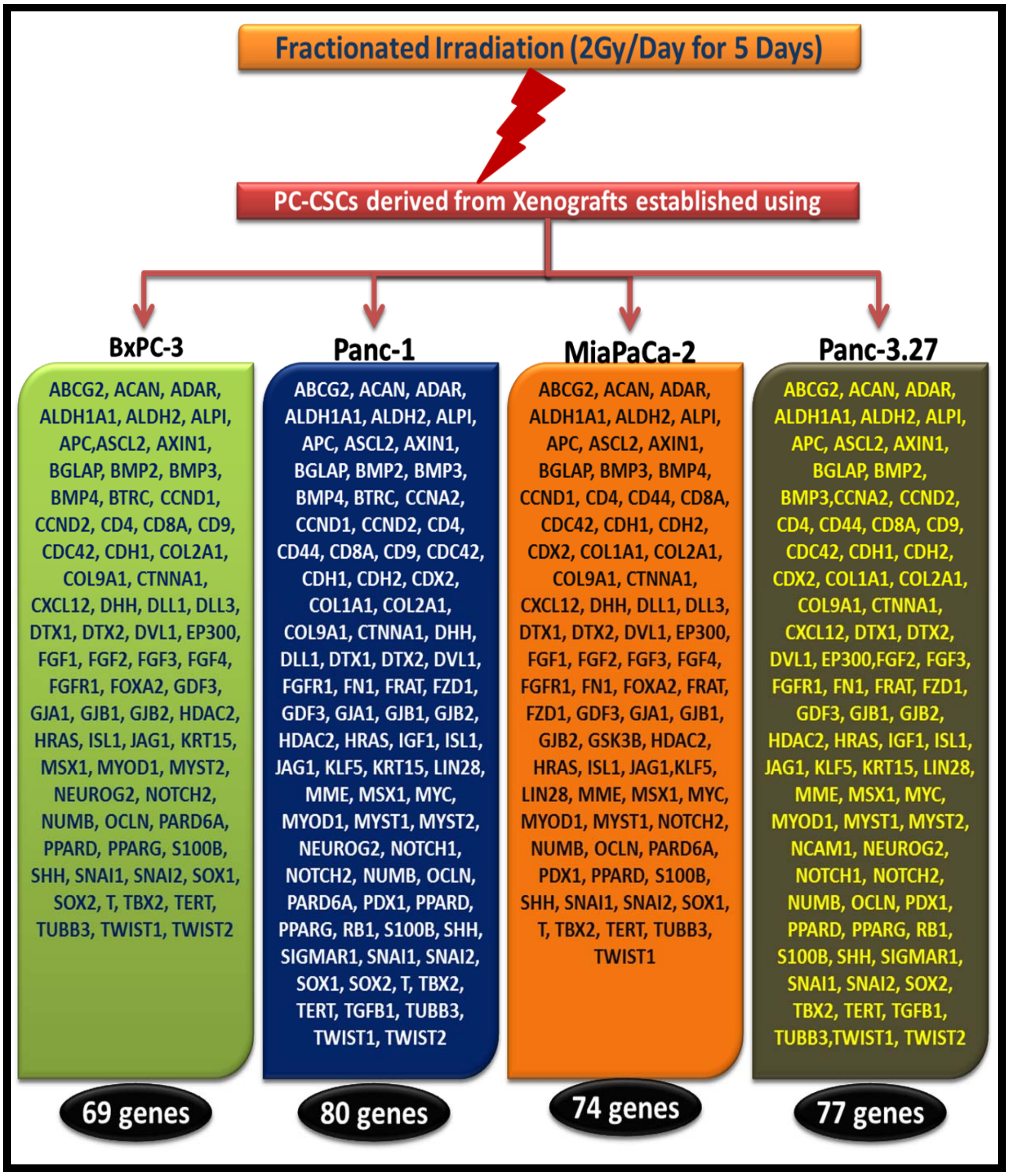

Additional File 2. Clinical doses of radiation (2Gy/Day for 5 days, for a total dose of 10Gy) significantly induced (≥2 Fold upregulation) EMT and stem cell-related transcriptome in PC-CSCs derived from Panc-1, Panc-3.27, MiaPaCa-2, or BxPC-3 cells' established xenografts. Forty-three genes, including *ABCG2, ACAN, ADAR, ALDH1A1, ALDH2, ALPI, APC, ASCL2, AXIN1, BGLAP, BMP3, CD4, CD8A, CDC42, CDH1, COL2A1, COL9A1, CTNNA1, DTX1, DTX2, DVL1, FGFR1, GDF3, GJB1, GJB2, HDAC2, HRAS, ISL1,JAG1, MSX1, MYOD1, NOTCH2, NUMB, OCLN, PPARD, S100B, SHH, SNAI1, SNAI2, TBX2, TERT, TUBB3,* and *TWIST1* showed consistent *cell-line independent* upregulation.
