# Supplementary material for: Polyphenols from marine brown algae target radiotherapy-coordinated EMT and stemness-maintenance in residual pancreatic cancer
Source: Stem Cell Res Ther. 2015 Sep 22;6(1):182. doi: 10.1186/s13287-015-0173-3 (PMC4578749; doi:10.1186/s13287-015-0173-3)
Supplement: Additional file 6: Figure S6. — Showing representative microphotographs from N-Cadherin-stained PC TMA constructed with xenografts (established from MiaPaCa-2) exposed to mock irradiation or fractionated irradiation, with or without SA-EA, PT-EA, and HT-EA fractions. Pullout shows the staining pattern (20× magnification). (PDF 3032 kb) [file 13287_2015_173_MOESM6_ESM.pdf]

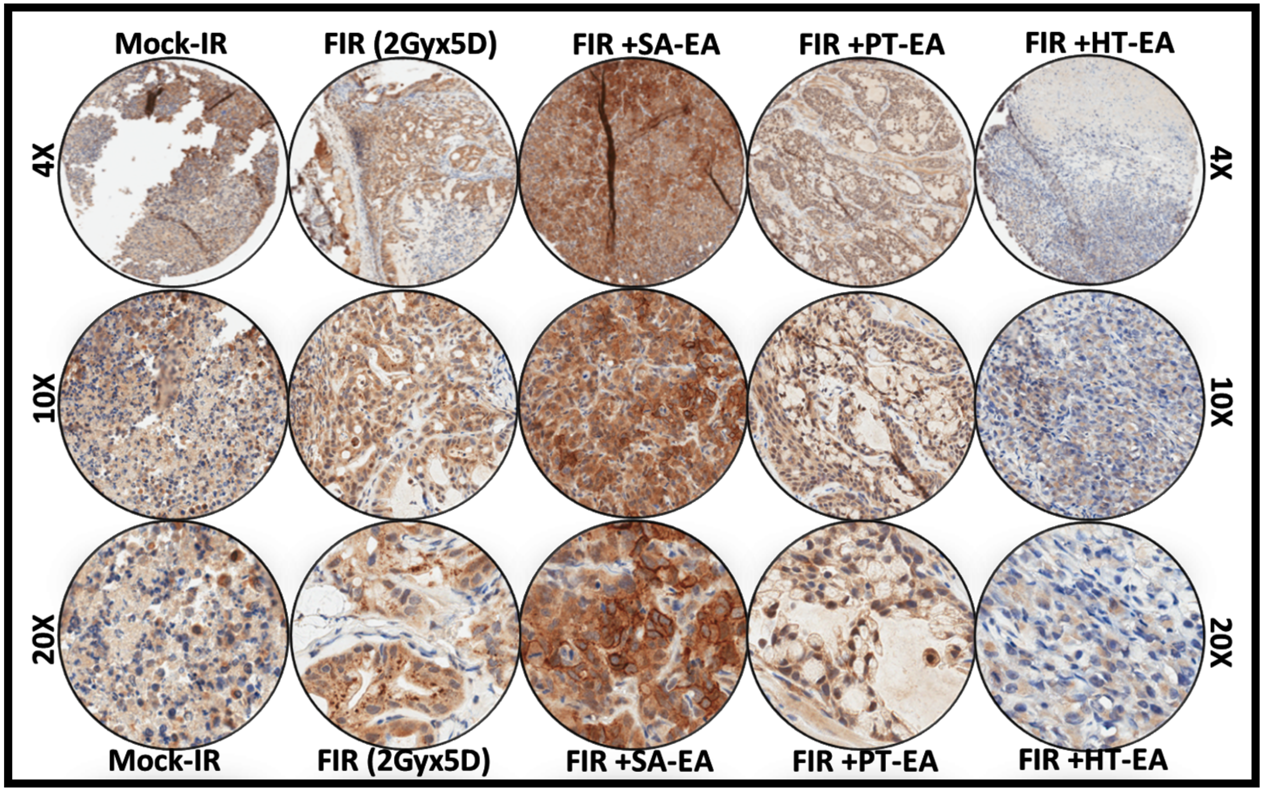

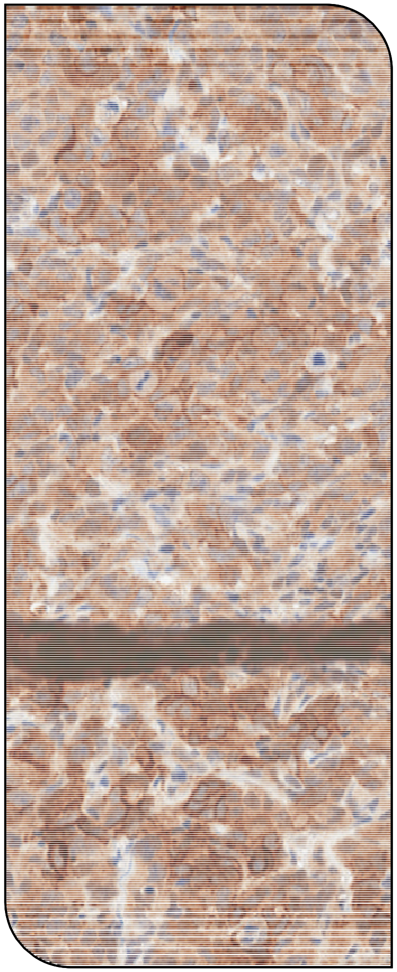

Additional File 6. Representative microphotographs from ***N-Cadherin***-stained PC tissue micro array (TMA) constructed with xenografts (established from ***MiaPaCa-2***) exposed to mock-irradiation or fractionated irradiation, with or without SA-EA, PT-EA, and HT-EA fractions. ***Pullout*** shows the staining pattern (20x magnification).
